# Supplementary figures and images for: Mechanosensitivity is an essential component of phototransduction in vertebrate rods
Source: PLoS Biol. 2020 Jul 15;18(7):e3000750. doi: 10.1371/journal.pbio.3000750 (PMC7384764; doi:10.1371/journal.pbio.3000750)

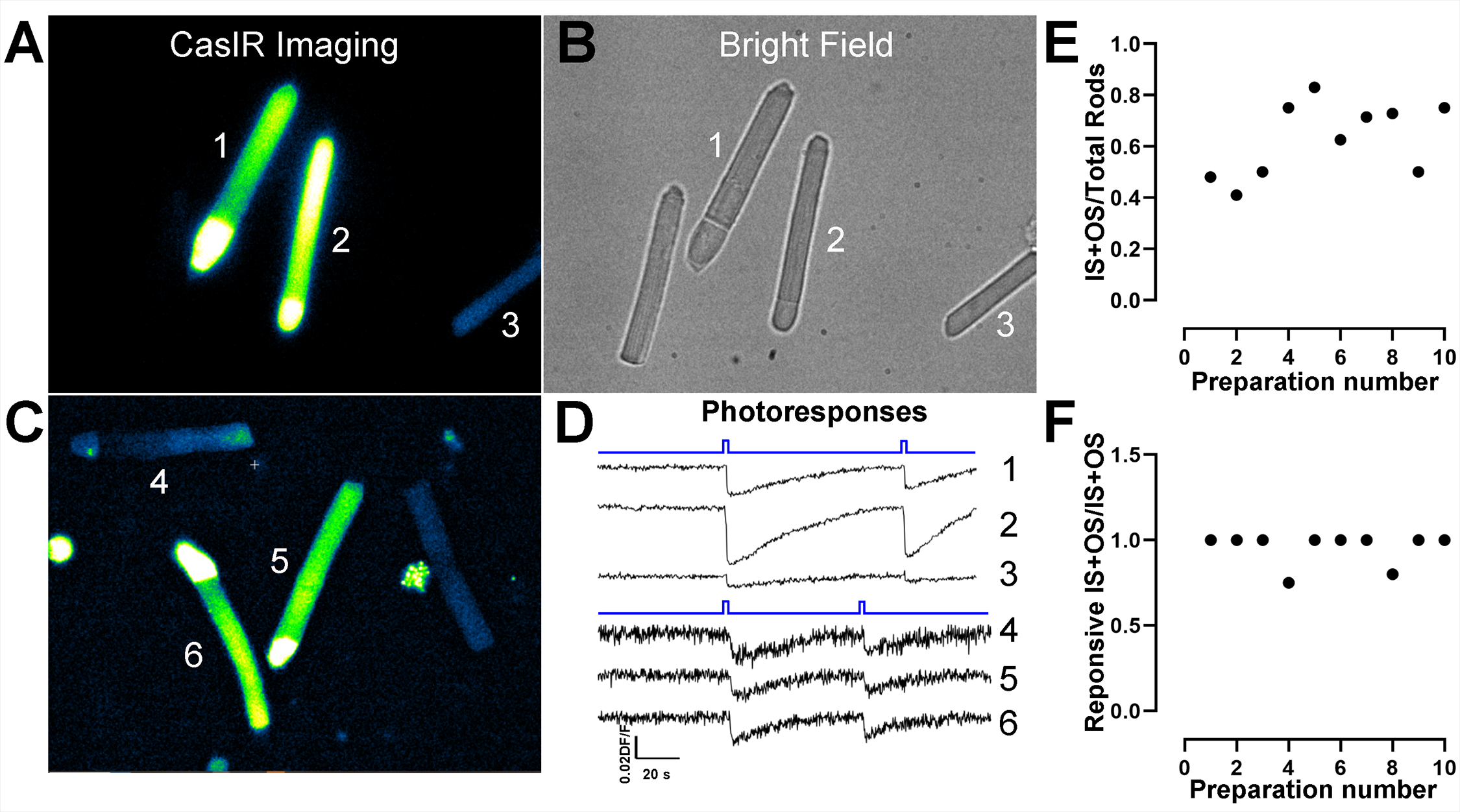

Supplement: S1 Fig — (A and C) Images of the fluorescence emitted by the calcium fluorescent dye CaSiR from rods in the preparation. (B) is a bright-field image obtained with a light source at 750 nm corresponding to the fluorescent image in A. Rods were viewed with a 60× objective, and of the 4 rods viewed in B, 3 were composed of IS + OS (labeled 1, 2, and 3). (D) Photoresponses obtained from the IS + OS shown in A and C. The blue traces are the light stimulus monitor; flash intensity was equivalent to about 104 R*. Numbers near the traces in D were obtained from the IS + OS with the same number in A and C. (E) Fraction of IS + OS rods out of the total number of rods in 10 representative preparations (S5 Data). (F) Fraction of IS + OS rods from which photoresponses could be measured out of the total number of ISs + OSs in that preparation (S6 Data). These fractions from each preparation were obtained from 10 random fields of view such as those shown in A–C. CaSiR, Calcium ion detecting probe based on silicon rhodamine; IS, inner segment; OS, outer segment. (TIF) [file pbio.3000750.s001.tif]

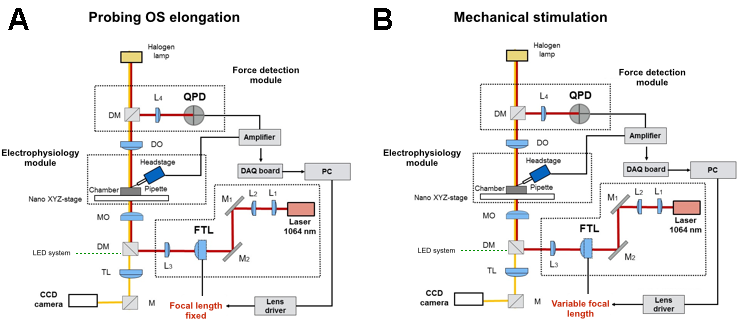

Supplement: S2 Fig — 1, inverted microscope; 2, OOT; 3, force measurement module. Optical components: L1, L2, convergent lenses, f1 = f2 = 100 mm; M1, mirror; FTL, fFTL = 55–90 mm; FL, f = 150 mm; DM1 (900 dcsp; Chroma, Bellows Falls, VT, USA); DM2 (XF22045, Chroma); TL; MO, Olympus 60×, NA 1.4, oil immersion; DO, 10×, NA 0.3; DM3 (900 dcsp, Chroma); L3, convergent lens, f = 40 mm. dcsp, Dichroic ShortPass; DM, Dichroic Mirror; DO, condenser objective; FL, Fixed focal Lens; FTL, Focused Tunable Lens; MO, Microscope Objective; NA, numerical aperture; OOT, oscillatory optical trap; QPD, quadrant photodetector; TL, Tube Lens (TIF) [file pbio.3000750.s002.tif]

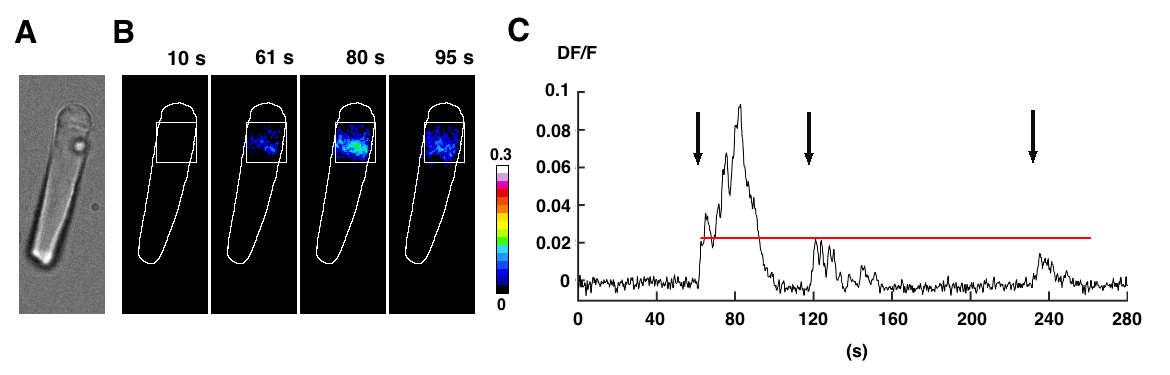

Supplement: S3 Fig — (A) Trapped bead in contact with the base of the rod OS under bright-field IR imaging. (B) Fluorescence change (DF/F) images, showing the ROI (white box) used to quantify the fluorescence change versus time before and during the first mechanical stimulation. (C) Calcium transients evoked by the repeated mechanical stimulations (indicated by the dark arrow). The amplitude of the first and fast calcium transient (indicated by the horizontal red line) is reproducible, while the second and larger component declines. IR, infrared; OS, outer segment; ROI, region of interest (TIF) [file pbio.3000750.s003.tif]

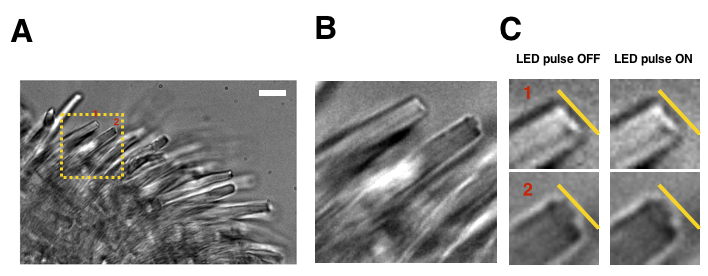

Supplement: S4 Fig — (A) A bright-field view of a piece of retina under IR light at 750 nm. (B) Zoom of the yellow dotted box in A. (C) Zoom of the tips of OS in the yellow dotted squares before illumination, during illumination, and after 20 s. The light-induced shortening of the rod OS corresponds to 2–4 pixels: given that a pixel corresponds to approximately 120 nm, the shortening is on the order of 200–400 nm. The enclosed video provides additional support to the light-induced OS shortening. IR, infrared; OS, outer segment. (TIF) [file pbio.3000750.s004.tif]

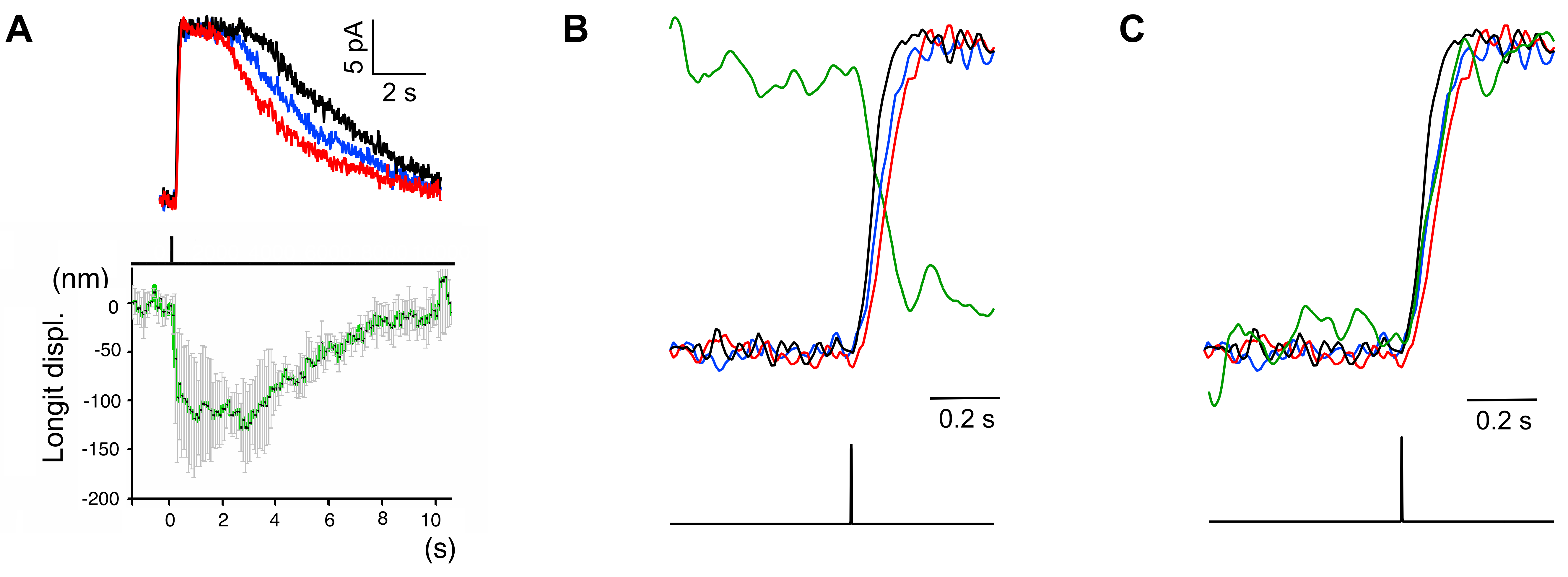

Supplement: S5 Fig — (A) Upper panel, 3 photoresponses to flashes of light equivalent to about 0.5, 1, and 2.5 × 10^4^ R* (red, blue, and black traces, respectively); lower panel, time course of shortening evoked by a flash of light at 491 nm, equivalent to about 104 R*. (B) As in A, but on a more expanded timescale; (C) superposition of all these traces after normalization of the maximum to 1. Traces in the upper panel of A were obtained from the same cell, and the trace in the lower panel of B was the average of 5 different experiments to the same light flash in different cells. OTs, optical tweezers. (TIF) [file pbio.3000750.s005.tif]

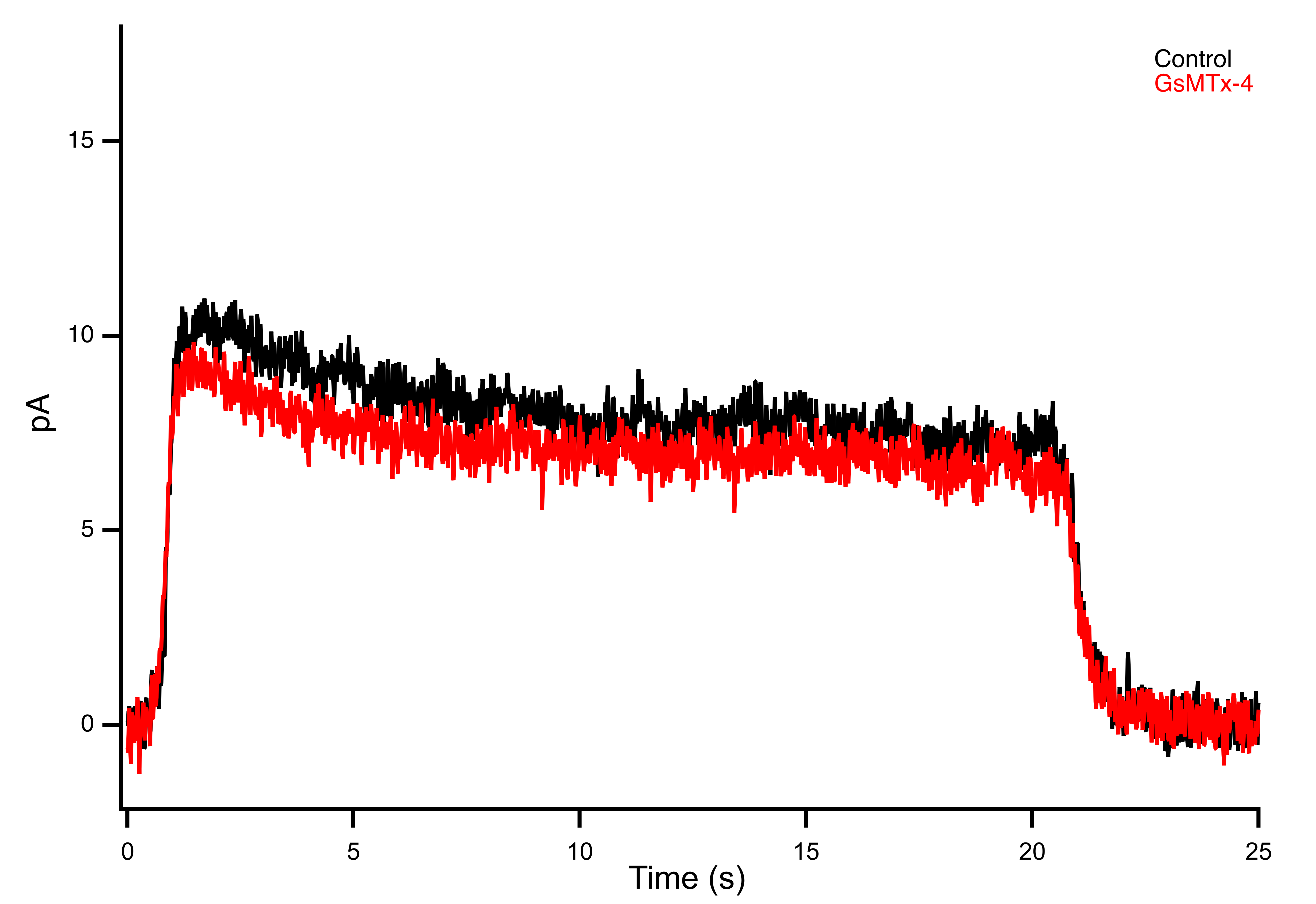

Supplement: S6 Fig — Maximal photoresponse to a saturating flash of light was 18 pA. GsMTx-4, M-theraphotoxin-Gr1a. (TIF) [file pbio.3000750.s006.tif]

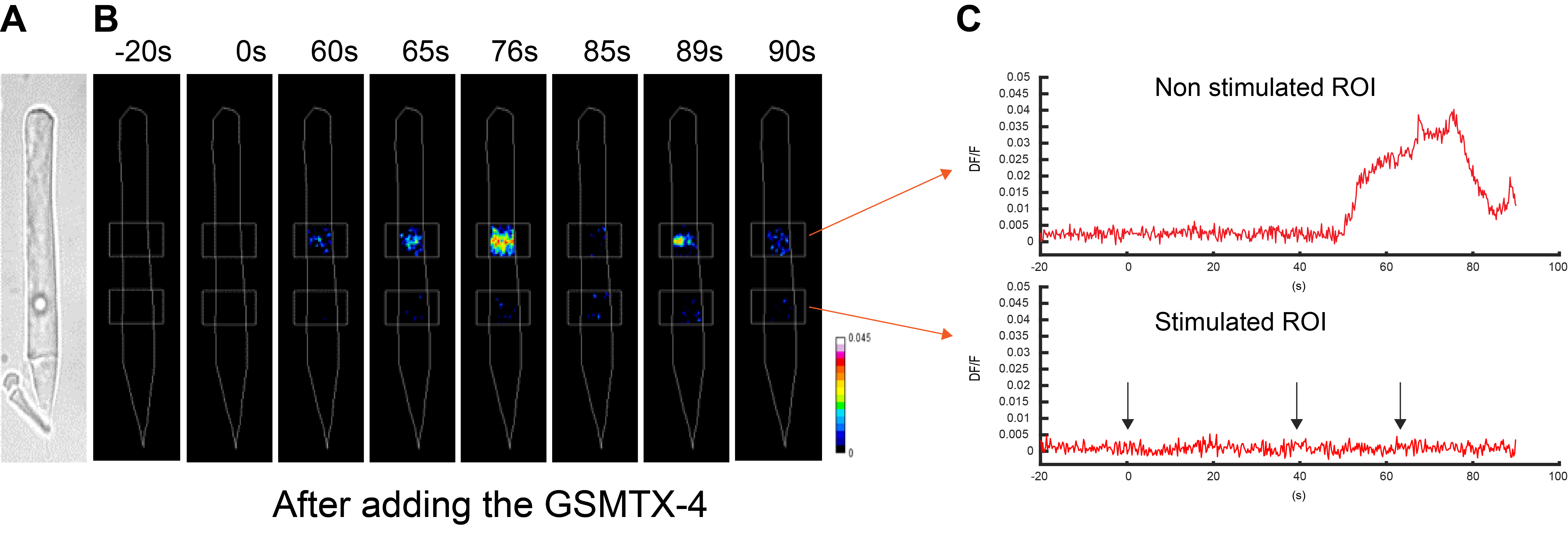

Supplement: S7 Fig — (A) A trapped bead in contact with the rod OS under bright-field IR imaging. (B) Fluorescence change (DF/F) images, showing the 2 ROIs used to quantify the fluorescence change versus time. (C) Time course of the evoked DF/F change from the 2 ROIs in B. Mechanical stimulation (black arrow) as indicated in B. In the presence of GsMTx-4, mechanical stimulation did not evoke any calcium transients, but spontaneous calcium transients could be observed. GsMTx-4, M-theraphotoxin-Gr1a; IR, infrared; OS, outer segment; ROI, region of interest. (TIF) [file pbio.3000750.s007.tif]

Marker

Retina

Empty

ROS

130

100

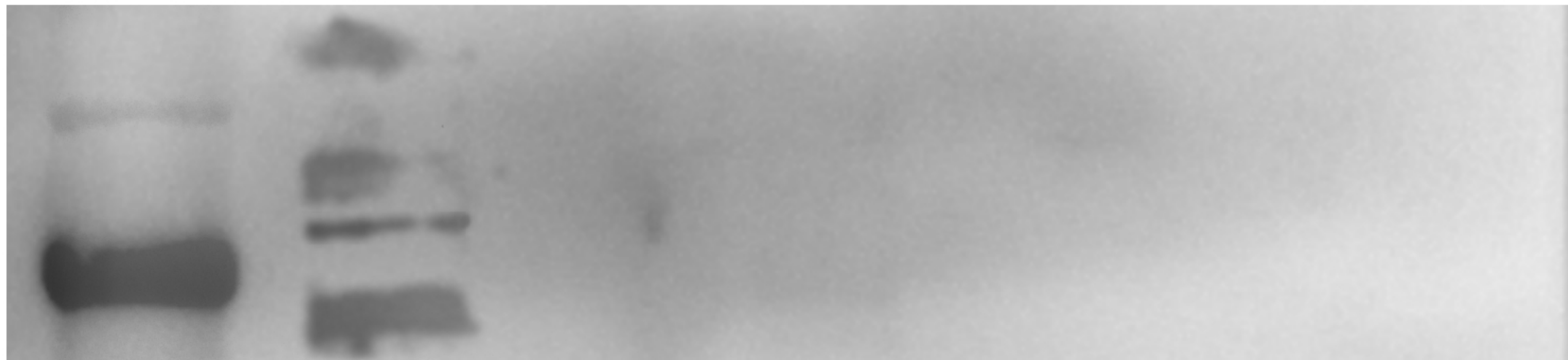

Supplement: S1 Raw Image — WB, western blot. (PDF) [file pbio.3000750.s016.pdf]
