# Supplementary material for: Mechanosensitivity is an essential component of phototransduction in vertebrate rods
Source: PLoS Biol. 2020 Jul 15;18(7):e3000750. doi: 10.1371/journal.pbio.3000750 (PMC7384764; doi:10.1371/journal.pbio.3000750)
Supplement: Attachment — Submitted filename: ResponseReviewers.docx [file pbio.3000750.s017.docx]

-------- Original Message --------
Subject: Your PLOS Biology Submission (PBIOLOGY-D-20-00239R1) - [EMID:ba336080562bef2c]
Date: 2020-02-29 02:07
From: "PLOS Biology" [<em@editorialmanager.com>](mailto:em@editorialmanager.com)
To: "Simone Mortal" [<smortal@sissa.it>](mailto:smortal@sissa.it)
Reply-To: "PLOS Biology" [<plosbiology@plos.org>](mailto:plosbiology@plos.org)

Dear Dr Mortal,

Thank you very much for submitting your manuscript "Rod photoreceptors require mechanosensitivity for optimal phototransduction" for consideration as a Research Article at PLOS Biology. Your manuscript has been evaluated by the PLOS Biology editors, an Academic Editor with relevant expertise, and by four independent reviewers.

In light of the reviews (below), we will welcome re-submission of a much-revised version that takes into account the reviewers' comments. We'd like to ask you to address the concerns raised by reviewers 1, 2, and 4 fully, particularly the effect of the 650 nm light exposure on the rods. You will need to respond to reviewer 3's criticisms conscientiously and, as an essential requirement, please comment comprehensively on the quality of your preparation mentioned by this reviewer who points out the fact that frogs rods preparation is very robust. We cannot make any decision about publication until we have seen the revised manuscript and your response to the reviewers' comments. Your revised manuscript is also likely to be sent for further evaluation by the reviewers.

We expect to receive your revised manuscript within 2 months.

Please email us ([plosbiology@plos.org](mailto:plosbiology@plos.org)) if you have any questions or concerns, or would like to request an extension. At this stage, your manuscript remains formally under active consideration at our journal; please notify us by email if you do not intend to submit a revision so that we may end consideration of the manuscript at PLOS Biology.

Answers to the Editor:

**1 - the effect of the 650 nm light exposure on the rods**.

The steady light at 650 nm used to excite CaSiR-1 was approximately equivalent to about 500 R*/rod/s and reduced the circulating dark current by approximately 40%.

This is now stated at page 6 of the manuscript. We apologize for not having given this important information.

**2 - the quality of your preparation mentioned by this reviewer who points out the fact that frog rods preparation is very robust.**

The figure, here below, shows representatives image of our preparation in bright field (B) and in fluorescence (A and C) emitted by our dye CasIR-1. More than 30% of the isolated rods are composed of an inner (IS) and outer segment (OS) where the IS is bright, and the base of the OS is dark and its tip more luminous. These IS+OS are functional rods as they exhibit a light response (measured as a change in fluorescence) as shown below:

Fig. 8: The quality of the preparation. A and C: images of the fluorescence emitted by the Calcium fluorescent dye CasIR from rods in the preparation. B is a bright field image obtained with a light source at 750 nm corresponding to the fluorescent image in A. Rods were viewed with a 60X objective and of the four rods viewed in B three were composed of IS+OS (labeled 1,2 and 3). D: photoresponses obtained from the IS+OS shown in A and C. The blue traces are the light stimulus monitor; flash intensity was .equivalent to 2.5 10^4^ Rh* Numbers near the traces in D were obtained from the IS+OS with the same number in A and C. E: fraction of IS+OS rods out of the total number of rods in 10 representative preparations. F: fraction of IS+OS rods from which photoresponses could be measured out of the total number of IS+OS in that preparation. These fractions from each preparation were obtained from 10 random fields of view such as those shown in A-C.

The quality of the preparation varies with the animal and the skill of the experimenter as shown in panel E of the above figure, but most of IS+OS rods are functional, varying from 75 up to 100 %. This figure has been added as a new figure in the Methods section.

From our previous experience, toad rods (Bufo marinus and Bufo bufo) are rather fragile, but rods from the tiger salamander (Ambystoma tigrinum) are much more robust. We have observed that rods from Xenopus laevis frogs properly dissociated are often functional. In addition, Xenopus laevis frogs are easier to keep than tiger salamanders, and they also reproduce well in captivity. These are the reasons we have opted to work with Xenopus laevis rods.

RESPONSE TO REVIEWS:

We are very grateful to all four reviewers for their positive and constructive reviews. We have dealt as comprehensively as we possibly could with their suggestions, and we trust that the Editor and reviewers will agree that we have incorporated changes to address almost all of their concerns, and that as a result the paper is substantially improved.

For many of the reviewers’ concerns, it was straightforward either to revise Figures or to re-word the text and, as you requested, we set out below our responses to their comments and we list the changes that we have made.

In a few cases, though, additional experiments were requested, and we immediately began tackling these. For three of the requested additional experiments, we have been able to conduct two repetitions of each experiment, and found that they support our interpretation, and we describe these in our detailed responses. However, with the COVID-19 situation in Italy, we are currently unable to perform additional experiments, and realistically it could be 6 months before we might be in a position to start experiments again. Rather than delay our report by what would inevitably be a period of greater than 6 months, we wish to request that the reviewers consider our revised paper and our detailed responses with some allowance for the conditions that are imposed by the current pandemic.

Reviewer #1: This manuscript by Bocchero and colleagues reports novel and interesting investigations of the mechanosensitivity of rod photoreceptors of the marine frog Xenopus laevis.  The study combines electrophysiology, biochemistry and optical tweezers to provide evidence for the following observations:
 (1) The mechanosensitive channels TRPC1 and Piezo1 are expressed in Xenopus rod photoreceptor inner and outer segments.
(2) Rod receptors (both outer and inner segments) respond to mechanical stimulation by local transient increases in cytosolic calcium.
(3) Inhibitors of these mechanosensitive channels decrease the duration of bright but not dim electrical flash response.
(4) Rod outer segments shorten in response to bright light flashes.
(5) Mechanosensitive channel proteins have significant homology with other protein families known to be involved in vertebrate phototransduction.
The authors posit that mechanotransduction in photoreceptors is fundamentally important in regulating bright light response kinetics in rod photoreceptors and may have important implication for regulation of outer segment homeostasis by providing mechanisms important for regulation of outer segment renewal.
The studies are carefully designed, and by their multidisciplinary nature, effectively address the issues of mechanosensitivity of rod photoreceptors.  The experiments and data are clearly described and illustrated, and the results that are reported are compelling.  Conclusions reached are justified by the nature and quality of the data presented.  Of particular importance is the novelty and importance of the findings for basic understanding of vertebrate phototransduction.

We appreciate the careful reading of the MS and the positive comments of the Reviewer.

Specific comments and questions are detailed below:
Page 2, para 2: The authors provide background information in the introduction that the mechanosensitive channels TRPC and Piezo are found in a range of other tissues where they modulate control of osmotic equilibrium and sensitivity to mechanical stimulation. What is missing here is a more complete discussion of the story of TRP channels and their relationship to vision, particularly in invertebrate photoreceptors.  Here, these channels have been shown to be calcium permeant, and activated as a part of the PLC/IP3 signaling cascade.   Their calcum permeability is likely important as the authors report here that local mechanical stimulation of rods produces calcium transients.  The authors should discuss briefly this history, starting in 1969 when Cozens and Manning first reported mutants (TRP) in which flash responses were abnormally transient.  Since that time a wealth of electrophysiological, biochemical, and genetic studies have established up to 28 different mammalian
isoforms of TRP that regulate a wide range of cellular process that include mechanical sensitivity.

We have inserted the following paragraph in the Introduction:

**Transient receptor potential (TRP) channels** form a superfamily of cation-selective ion channels located in cell membranes, that are involved in various sensory modalities, such as chemoreception, thermoreception, mechanoreception, and photoreception. TRP channels were discovered more than three decades ago in photoreceptors of fruit flies ([Drosophila](https://www.britannica.com/animal/Drosophila)) (for review, see Minke and Cook, 2002; Clapham, 2003; Montell, 2005; Myers et al., 2008; Bohlen et al., 2011). The TRP super-family in animals contains at least seven families, comprising around 30 sub-families, and the majority of these isoforms are expressed in vertebrates. Vertebrate TRPC channels (C for canonical) are so-named because they are most closely related to the canonical TRP channels involved in *Drosophila* phototransduction. There are seven sub-families of vertebrate TRPCs, of which TRPC1, 4 and 5 are very closely related, as will be considered subsequently.

Page 4, Figure 1 legend: "scale bar 100μm" cannot be correct.  Please check and modify

We have corrected the scale bar and the correct scale bar is 50 μm.

Page 5 para 2: "….basal end appearing dark."  I suggest that at an appropriate location in the manuscript (perhaps Methods) there be included a statement regarding the relationship between color in the images of rods and relative calcium concentration.  To state that one end of the cell is "dark" is not sufficiently clear.

We have inserted a scale bar to the color images of rods and we have clarified this issue in the text by inserting the following paragraph:

“The emitted fluorescence of the CasIR dye at the OS tip of IS+OS was usually 4 times larger than at its base so that the base appears darker than the tip”

Page 6, para 2: "DF/F" should be precisely defined (in Methods).

We have defined DF/F in the Methods section and indeed DF/F = (F(t=0) – F(t))/F(0) where F(0) is the fluorescence intensity at the beginning of the experiment and F(t) is the fluorescence intensity at time t. In panels B, F and L of Figure 2, the color maps indicate DF/F after spatial averaging over a window of 3x3 pixels. In subsequent panels (C, D, G, H, and M, N), DF/F was computed over the region indicated by the white boxes in panels B, F and L. This information has been added to the legend.

Page 6, para 2 (bottom of page):  In the third line from bottom of page calcium is described as being released from internal stores.  A few lines later channels are referred to as the means by which cytosolic calcium increases. The implicit assumption seems to be that calcium is released from the discs through membrane channels.  However, the authors provide no evidence to argue that this is be the case.  Could, for instance, the calcium come into the cell from the outside through channels in the plasma membrane?

We have revised the text (Page 6-7) to indicate that the calcium could have come either across the plasma membrane or from internal stores, or from both. In the case of the OS, “internal stores” encompasses possible fluxes from within the disks.

Page 10, figure 4: "thanc 1s". (?)

We have corrected this mistake and the correct word is “than 1 s”.

Page 15, para top: The authors present evidence (Figure 6 and legend) that TRPC1 and Piezo 1 channels are found in the inner segment/ellipsoid and data is presented that mechanical stimulation of the IS results in calcium transients. They show also that calcium transients can also be elicited in outer segments, but there is no convincing demonstration that either of these channel types exist there by immunocytochemistry. The argument that some other MSC channel type, as yet unidentified, is responsible, is weak. I am not sure what should be done here to deal with this logical gap.

We have addressed this issue with a new paragraph in the Discussion.

Page 20, para 2.  "…between and Actin 22 and 24 C".  Please clarify and/or correct.

We have corrected this mistake and the correct sentence is “.. between 22 and 24 ºC”

P21, para 2. "DF/F" should be defined more precisely.

We have defined DF/F properly in the Methods section as:

“DF/F = (F(t=0) – F(t))/F(0) where F(0) is the fluorescence intensity at the beginning of the experiment and F(t) is the fluorescence intensity at time t. The fluorescence analysed in the panels C, D, G, H, M and N of Fig.2 was computed either in the large region indicated by the white boxes in Fig2B, F and L. In panels B, F and L the color maps indicate DF/F computed at each pixel within the white box after a spatial averaging in a window of 3x3 pixels.”

Page 26 Fig SI2 legend: This legend and its associated figure illustrates the effect of repeated mechanical stimulations. The legend states: "The amplitude of the first and fast calcium transient is reproducible, while the second and larger component declines."  On the face of it, this suggests that any mechanical stimulation may cause a change in the cell's response to subsequent mechanical stimulations.  What then is the response of the cell to the attachment of a bead?  Does this not perturb the channels so as to confuse the effects of stimulations subsequent to bead attachment.  Some discussion of this issue would be helpful.

We have addressed this issue in revised text (Page7, together with an additional Figure, as follows:

Text revision (Page7):

“In several experiments we repeated the same mechanical stimulation at least three times, and we observed a decline in the magnitude of the response (Fig. SI 2); it is possible that an initial fast component remained unchanged and that a second larger component declined, but this is not clear from the results. To determine whether a similar phenomenon could have been initiated by attachment of the bead, we also conducted experiments monitoring the calcium signal along with bead position and force measurement during bead attachment (Fig. SI 3). We conclude from these experiments that when the bead attaches to the membrane there is no major response or reaction in the cell.

**Additional Figure:**

**Figure 9**: A: Voltage command to the Focus Tunable Lens (FTL), controlling the vertical displacement of the optical trap. The steps in the dotted lines show the lowering of the bead before adhesion and raising of the bead after mechanical stimulation. B: Force recorded in the vertical direction by the QPD. C: Fluorescence signal DF/F monitoring the level of intracellular calcium. There was no change in DF/F at the time of the adhesion of the bead (marked by a decrease of noise in the force trace), but DF/F increased following the mechanical stimulation, indicated by “Stim.”.

Page 26, para 3 (video imaging):  This legend and associated figure describes changes in outer segment length with light stimulation.  Was this experiment performed in the presence of the channel inhibitor?  If this experiment is described, I missed it.  If not, this result would provide an interesting addition

The Reviewer is correct and we have followed his suggestion and we have repeated the experiment in the presence of GSMTX-4, the inhibitor of MSCs.

We have verified that in the presence of GSMTX-4 rods still shrink in response to a bright flash of light, but we could not determine whether this shrinkage is to some extent regulated or modulated by GSMTX-4 and MSCs.

Reviewer #2: The manuscript by Bocchero and colleagues describes experiments aimed at studying mechanosensitivity in Xenopus rod photoreceptors and its possible link to phototransduction. The authors begin by demonstrating, with the help of a calcium dye and optical tweezers, that mechanical stimulation of isolated functional rods results in local increase in calcium. They next demonstrate that bright flash stimulation in isolated rods causes slight shortening of their outer segments, and that application of the mechanosensitive channel blocker GsMTx-4 produces a slight reversible acceleration of the saturated photoresponse but does not affect the kinetics of the dim-flash response. Finally, the authors perform gene association analysis and find that TRPC1 and Piezo1 are both closely associated with phototransduction proteins and are expressed in the rod inner segments of Xenopus. Based on these results, the authors suggest that mechanosensitivity is required for optimal
phototransduction in rods.

The study is original and interesting, and uses state of the art optical tweezers tools. The findings are intriguing but, ultimately, their relevance for normal (or optimal) phototransduction in rods is not very clear.

Specific essential issues that should help address this and strengthen the study and its impact include:
-    The authors perform a series of negative control experiments to rule out artifacts in their experiments linking calcium and mechanotransduction in rods. However some key control experiments are missing.

We appreciate the careful reading of the MS and the positive comments of the Reviewer.

Specifically:
o    It is not shown whether "unresponsive" rods or truncated rod outer segments change their length or calcium levels in response to photostimulation. If mechanosensation is linked to calcium and phototransduction, presumably they should not.

As we explained in the manuscript, “unresponsive” rods are defined as those that showed no detectable change in calcium level in response to photostimulation. Likewise, we have not seen any drop in intracellular calcium in truncated outer segments. We have now attempted new experiments with OT on “unresponsive” rods, and in two experiments we were unable to detect any shortening, though to date we have not been able to set the detection limit at less than 50 nm.

Similarly, an important negative control experiment with light-adapted or bleached functional rods is also missing.

We have also attempted experiments with OT on light-adapted rods. In two such experiments we were unable to detect any shortening, though again we can only set the detection limit at 50 nm from those experiments.

o    It is not shown whether application of the MSC blocker blocks the light-induced shortening of the rod outer segments.

The Reviewer is correct and we have followed his suggestion and we have repeated the experiment in the presence of GSMTX-4, the inhibitor of MSCs.

We have verified that in the presence of GSMTX-4 rods still shrink in response to a bright flash of light, but we could not determine whether this shrinkage is to some extent regulated/modulated by GSNMTX-4 and MSCs.

o    Finally, it is not clear how the authors rule out effects of GsMTx-4 on the mechanosensitive channels from its possible effects on the phototransduction CNG channels of cascade.

We have addressed this issue by attempting additional experiments on the cGMP-activated current recorded from excised patches of rod outer segment membrane. In two such experiments we were unable to detect any change in the size of the induced currents, within a detection limit (to date) of a 10% change.

-    An obvious omission from the study is an experiment on the possible effect of mechanotransduction-induced calcium increase on rod light adaptation. If the light-induced shortening of the rod outer segment is associated with increase in calcium, it should counteract the light-driven decline in calcium in the course of phototransduction, and should therefore, partially suppress light adaptation. A light-adaptation experiment with vs. without the MSC blocker GsMTx-4 might be able to address this question.

We have addressed this question using suction pipette experiments, with application of a steady adapting background, either under control conditions or during application of GSMTX-4. The results from one of two such experiments are illustrated in SI 6, where we found no reliable change in the red trace (in GSMTX-4) versus the black trace (control). Although the red trace is very slightly smaller than the black trace, this might readily be explicable in terms of a slight rundown in the cell’s circulating current. But, as far as we could tell, the rising phase of the response and the sag to a plateau level were essentially unchanged.

Figure SI 6: Comparison of the response to a step of light of 20 sec duration and equivalent to about 250 Rh*/sec in control conditions (black) and in the presence of GSMTX-4 (red) from the same cell. Maximal photoresponse to a saturating flash of light was 18 pA.

-    The range of length changes observed in rods is on the order of 100-200 nm - a miniscule shortening effect considering the 50-60 µm length of the rod outer segments in Xenopus. Furthermore, it is not evident that even this small change in length occurs in the intact retina in vivo, when photoreceptors are mechanically bundled together and meshed with the apical processes of the RPE cells in a tightly-packed structure.

The reviewer is correct that the length changes are very small, as a fraction of the overall length of the outer segment. However, that is not the point we are making, which is instead that the rapid shortening is a signal (a reflection, if you like) of a physical phenomenon that accompanies the light response. Furthermore, the change in length that we observe is to some extent limited by the fact that the cells are adhering to a surface. By using video imaging of unconstrained cells, we reported shortenings of 200–400 nm (Supplementary Movie 1; see also new Figure 4). These measurements are broadly consistent with the previous experiments of Lu et al. (2017, 2018) which showed shortenings of 400–600 nm in unconstrained OS of leopard frogs (*Rana pipiens*); they also showed similar movements in the intact living retina. The importance of our current approach using OT is that it is able to resolve these tiny movements with very high temporal resolution, of the order of milliseconds. We have revised the text on page 10 to emphasize these points.

Minor issues:
-    The authors mention disc shedding in the Introduction and in the Discussion but it is not clear or discussed how this process might be related to mechanosensation.

We have revised the Introduction and Discussion to remove mention of disc shedding.

-    The authors should consider showing the effect of the 650 nm light exposure used for calcium imaging on the level of calcium in functional rods (as in Fig 1B), similarly to the effect shown on the saturating photoresponse in Fig. 1F.

Although it would in principle be nice to determine the absolute level of calcium during the 650 nm light exposure, we are not set up for such experiments, which would require ratiometric calcium measurements using Fura-2 or a similar dye. Furthermore, such an approach is “one shot” as the intense exposure to short wavelength light completely saturates the rods. And, as the calcium level during the 650 nm illumination would inevitably be lower than in darkness, the signal-to-noise would be even poorer than in published experiments on the dark level.

-    A number of words throughout the MS are split by a hyphen, e.g. pho-ton, re-duction…

We have checked the hyphens in the revised version of the MS.

Reviewer #3: This ms reports that small mechanical stimulation of the inner segment and outer segments of isolated Xenopus rod photoreceptors induce changes in intracellular calcium and subtle changes in the cells' responses to bright flashes. Because the spider toxin GSMTX-4 abolishes both the force-driven calcium channels and light-induced changes in cell volume, the authors infer that these channels are responsible for both of these phenomena. However, direct evidence that frog rods express any mechanosensitive channels are not convincing. The proximity of TRPC genes to those of other families of phototransduction genes is perhaps interesting in the larger context of the evolution of vision but not relevant to determination of a functional role of these channels in phototransduction.

More specifically, several different aspects of the manuscript raise questions:

Most (n>40) of the most-nearly intact rods used for calcium imaging had high levels of fluorescence and were unresponsive; far fewer cells (n=10) were deemed functional (p.5). This seems surprising given the typical robustness of rod photoreceptor preparations in cold blooded vertebrates and does not evoke confidence in the preparation.

We regret that our wording appears to have misled the reviewer. As stated in the text, the number of responsive rods was >30 rather than 10; i.e. we said “(Fig.1A right inset, and Fig.1B; n > 30)”. The value of “n=10” applied to the number of cells from which the trace in Fig. 1D had been averaged, and not to the number of responsive cells. Furthermore, we have found that as our skill with the technique has improved, the proportion of responsive cells has increased. Accordingly, we have reworded the text on page 5 to include more-recently recorded cells) as follows. In addition, we have added a new Figure summarising measurement from our recent trials.

“More-nearly intact rods, in which the outer segment remained connected to at least part of its inner segment (i.e. OS+IS), fell into two categories. On the one hand, we found one category of ‘unresponsive’ cells that showed no change in fluorescence upon exposure to blue light (Fig.1E). These cells were characterized by approximately uniform fluorescence along the OS, at a moderate to high level, and often showed a high level of fluorescence in the IS (Fig. 1E, n ˃80). A second category of cells exhibited a marked gradient of fluorescence along the outer segment, with the basal section fluorescing only weakly (Fig. 1A right inset, and Fig. 1B; n ˃50). Such cells exhibited a distinct drop in OS fluorescence in response to blue light (Fig. 1D, blue trace; n = 10; see also Fig. 8). We hypothesize that this second category of ‘responsive’ OS+IS represents functional rods, that...”

Fig. 8: The quality of the preparation. A and C: images of the fluorescence emitted by the calcium fluorescent dye CaSiR-1 from rods in two preparations. B is a bright field image obtained with a light source at 750 nm, for the same field as the fluorescence image in A. Rods were viewed with a 60X objective and of the four rods viewed in B three were composed of an IS+OS (labeled 1,2 and 3). D: photoresponses obtained from the IS+OS shown in A and C, with corresponding numbers. The blue traces are the light stimulus monitor. E: proportion of IS+OS as a fraction of the total number of rods in 10 representative preparations. F: proportion of IS+OS from which photoresponses could be measured as a fraction of the total number of IS+OS in that preparation. These fractions from each preparation were obtained from 10 random fields of view such as those shown in A-C.

Local mechanical stimulation using optical tweezers of both inner and outer segments caused transient increases in calcium that in 3 experiments were blocked by GsMTx-4. Although GsMTx-4 is considered a blocker of mechano-sensitive channels it inserts into lipid bilayers rather than blocks these channels directly. Could the calcium transients themselves have no molecular basis in mechano-sensitive channels per se and the toxin's actions be explained by changes in the fluidity of the membranes? Ways to more specifically assess and perturb channel localization and function are needed.

We are not aware of any evidence suggesting that GsMTx4 alters the fluidity of membranes, and nor is there evidence that it blocks channels directly. Instead, as we set out in the text, it is a gate modifier and is thought to act at the interface between MSCs and the membrane. We have revised the text to read:

“According to Gnanasambandam et al (2107), GsMTx4 is stabilized by lysine residues, and occupies a small fraction of the surface area in unstressed membranes. When applied tension reduces lateral pressure in the lipid phase, those residues penetrate deeper, acting as “area reservoirs”, leading to partial relaxation of the outer monolayer, thereby reducing the effective magnitude of the stimulus acting on the gate of the MSC.”

The small light-driven change in outer segment length measured with optical tweezers (Fig. 3) is quite elegant but it appears that the average movement actually slightly precedes light onset (Fig. 3D).

It is conceivable that the change in outer segment length does indeed slightly precede the electrical response, because it is possible that this mechanical effect results from molecular changes in the membrane (e.g. movement of the PDE) rather than from osmotic changes. However, the precise timing has not yet been determined unequivocally, either in published results or in our own work. In our own work, we are not able to make simultaneous recordings of shortening and the electrical response, and therefore we have needed to make comparisons in different experiments where we have used the same flash intensity, but there may inevitably be differences between different cells. Furthermore, it is important to ensure that the signal filtering is the same for the different physical signals being measured. With these factors in mind, and within the resolution of our experiments, we have not found any reliable difference between the onset phase of the mechanical and electrical responses.

SI 4: comparison of the time course of the electrical response measured with suction pipette and shortening measured with OT. A: upper panel 3 photoresponses to flashes of light equivalent to about 0.5, 1 and 2.5 x 10^4^R*/rod (red,blue and black traces respectively); lower panel time course of shortening evoked by a flash of light 491 nm, equivalent to about 104 R*/rod. B: as in A but on a more expanded times scale; C superposition of all these traces after normalization of the maximum to 1. Traces in the upper panel A were obtained from the sale cell and the trace in the lower panel B was the average of 5 different experiments to the same light flash.

This figure has been added to the Revised version of the MS as a new SI figure.

For the validation using video imaging (Fig. 3 SI) the change in length is reported to have corresponded to 1-2 pixels or 100-300 nm. How is that possible to see with standard microscopy?

We have used an objective lens with 60X magnification, numerical aperture NA=1.4 and a Hamamatsu ORCA-D2 camera with sensor’s pixel size of 6.5 µm. Considering the light wavelength, λ= 750 nm, the lateral optical resolution is: r ~ 320 nm. The sensor resolution is given by the pixel size. We used binning 2x, thus one image pixel corresponds to about 220 nm on the sample (p= 6.5*2*1000/60 = 216.7 nm ~ 220 nm). The sensor resolution matches at the limit the optical resolution: 3p ~ 2p (matching condition: 3p ≥ 2p). The experiment consists in recording the image of a piece of retina before and after a flash of light, at 2 frames / second and observe potential retraction of the rods. One frame illustrating a piece of retina with rods is shown in Figure 4A. We measure the retraction of the rods indicated in figure with numbers from 1 to 6. For each rod we define a segment of the same length (L= 10 µm) along the rod axis (white lines) and we calculate the kymograph. The kymogaph shows how the intensity distribution along the segment varies in time (Figure 4B). This variation is directly related to a potential retraction of the rod. As one can observe from the kymograph, after the FLASH (indicated by the blue line) there is a clear shift of the intensity, Δ, corresponding to the retraction of the rod. We calculate the values for the shift Δ with subpixel precision. To do this we first select small regions of interest (white rectangles in Figure 4B) of size ROI= 14x20 pixels. Then, using bilinear interpolation we define a new ROI over a grid with 20 times more points (pixels), in each direction, than that of the original ROI (Figure 4C). We then plot the intensities along the green and red lines, corresponding to the dark and light (after flash). To determine the shift Δ, we calculate the quantity: A=∫[(Id(x-Δ)-Il(x)])^2]dx, where Id(x) and Il(x) represent the intensity profiles for dark and light regions, and Δ= k*p/20 , with k=1:40. The subpixel value of the shift Δ is given by k for which the quantity A is minimized (Figure 4D). We found that the the rods shifted by Δ > 240 nm (i.e. more than 1 pixel), except the rod 3 for which Δ = 120 µm. The maximum shift Δ max = 330 nm corresponds to the rod 6.

**Fig. 4: Light induced shrinkage of intact rods in pieces of retina**. A: One frame from the movie (SI) showing a piece of retina with rods. B: Kymogaphs calculated along linear segments at the tip of the rods labelled with numbers from 1 to 6 in A show a shift, Δ, in the intensity profile after the flash (blue line), confirming rod shrinkage. Small regions of interest ROI (white rectangles with size= 14x20 pixels) are selected and zoomed in the second row. C) Bilinear interpolation of one ROI over a grid 20 time bigger (280x400 pixels) than the original. D) Intensity profiles (green and dotted red) before and after flash. The shift Δ is calculated to minimize the difference between the intensity profiles (solid red and green lines).

The effect of the spider toxin on the kinetics of the photoresponses is apparent only for the very brightest flashes, and even then the effect is very small. How does this demonstrate a requirement "for optimal phototransduction" (title)?  What is the mechanism?

The Reviewer is correct and we have changed the title of the MS to

**Mechanosensitivity is an essential component of phototransduction in vertebrate rods**

In our view the fast light induced shortening of OS is a novel observation which suggests that light induced mechanical/geometrical changes of the OS are a constituent aspect of phototransduction. As indicated by Reviewer #2 we are studying in more details the role of mechanosensitivity in light adaptation.

The text descriptions and quality of the data of Fig. 6 are not standard. The staining for Piezo1 in the inner segments is deemed specific but the greater staining of TRPC1 in the outer segments is deemed likely to be autofluorescence. Why do the authors think that, and if there is such evidence for it being an artifact, why show it? I also don't understand the point of Panels D-E - to show lack of TRPC1c staining?  If TRPC1 is only in the inner segment and not outer segment, why did the mechanical pulses of Fig. 2 work for both inner and outer segments and be blocked by the spider toxin in both cases?

The Reviewer is correct and this issue is puzzling. We dedicated a lot of effort and energy to identify the molecular mechanism of mechanosensitivity in the OS. There are two possible explanations for that:

1 – in the OS there are MSCs different from those we have looked for.

2 – mechanosensitivity in the OS is not mediated by MSCs but by a direct mechanical action on the disks.

While we are confident that we have identified the molecular origin of mechanosensitivity of the IS we recognize that we have not been able to clarify precisely this issue in the OS.

Reviewer #4: This manuscript entitled "Rod photoreceptors require mechanosensitivity for optimal phototransduction" by Bocchero et al. demonstrates mechanosensitivity of amphibian (Xenopus) rod photoreceptors and explores its role in the electrical activity of the rods as well as the genetic correlates between the genes coding mechanosensitive channels and phototransduction proteins. The methodology of the paper is solid and well-chosen including a combination of electrophysiology, optical tweezers (OT) and biochemistry. The utilization of OT together with calcium imaging is a clever choice taken into account the resolution needed in revealing the mechanosensitive movements. These are technically challenging recordings that appear to have been very carefully carried out. The overall findings of the paper are novel, have clear merits and are of broad interest to a wide audience in visual neuroscience. I have comments mainly related to the improvement of the presentation.

Overall: The resolution of figures was borderline even for reviewing purposes.

We have improved the quality of figures and we hope that now they are adequate.

Figure 1: What is the estimated photoisomerization rate caused by the excitation light (650nm) in these recordings? It would be important to report and/or estimate this for the reader to know the physiological state of the cells in these recordings.

The number of estimated photoisomerization of the excitation light was equivalent to 500 Rh*/sec

Fig. 1A: It would be good to either show as inset the excitation and emission spectra and/or move the text "CaSiR…Ex:660" etc to the legend of the Fig.

We have moved the information on the emission and excitation to the legend of the figure

Fig.1B&C It would be good to show all rods on the same scale: currently there are

Scalebars (10 um) in each sub panel but the scaling varies a bit (cf scale bars). The same scaling and a single joint scale bar would be better.

We have followed the Reviewer suggestions as shown in the modified figure below:

Page 5: What is the distribution between "response" and "unresponsive" rods? Currently example cells are shown in Fig. 1 but there is no clear statements of the population data related to number of cells etc. It would be useful to understand this distribution. E.g. could you show the distribution of all cells in terms of the a parameter that defines gradient-type "responsiveness" in Fig 1?

The figure, here below, shows representatives image of our preparation in bright field (B) and in fluorescence (A and C) emitted by our dye CasIR. More than 30% of the isolated rods are composed by an inner (IS) and outer segment (OS) where the IS is bright and the base of the OS is dark and its tip more luminous. These IS+OS are functional rods as they exhibit a light response as shown below:

Fig. 8: The quality of the preparation. A and C: images of the fluorescence emitted by the Calcium fluorescent dye CasIR from rods in the preparation. B is a bright field image obtained with a light source at 750 nm corresponding to the fluorescent image in A. Rods were viewed with a 60X objective and of the four rods viewed in B there were composed by IS+OS (labeled 1,2 and 3). D: photoresponses obtained from the IS+OS shown in A and C. The red traces are the light artitfact. Numbers near the traces in D were obtained from the IS+OS with the same number in A and C. E: fraction of IS+OS rods over the total number of rods in 10 representative preparations. F: fraction of IS+OS rods from which photoresponses could be measured over the total number of IS+OS in that preparation. These fractions from each preparation were obtained from 10 random fields of view such as those shown in A-C.

The quality of the preparation varies with the animal and the skill of the experimenter as shown in panel E of the above figure, but most of IS+OS rods are functional varying from 75 up to 100 %.

From our previous experience toad rods (Bufo marinus and Bufo bufo) are rather fragile but rods from the tiger salamander (Ambystoma tigrinum) are much more robust. We have observed that rods from Xenopus laevis frogs properly dissociated are often functional in a greater proportion. In addition, Xenopus laevis frogs are easier to keep than tiger salamanders and reproduce rather well also in captivity. These are the reasons we have opted to work with Xenopus laevis rods.

Page 6: "semi-dark-adapted": what is the estimated equivalent background light corresponding to these conditions in isomerization rates? This would be very useful to estimate within a reasonable accuracy. "Semi-dark-adapted" is not now anyhow defined.

The steady light at 650 nm used to excite CaSiR-1 was approximately equivalent to about 500 R*/rod/s and reduced the circulating dark current by approximately 40%.

Fig 3C & D: It would be good to see standard deviation or SEM with the average trace. Now it seems that each individual trace is an average of two traces but it is unclear how many traces are averaged for the "grand" mean and what is the error bar. Shaded SEMs around the "grand" mean would work too.

We have modified the figure, as shown below, to follow the Reviewer’s suggestion.

Fig 4H: What are the units of the ordinate? pA? Please, indicate in the figure

We have modified the figure so to follow the Reviewer suggestion, as show below

Page 11 & 15: It is stated that the impact of GsMTx-4 is larger for saturating intensities. It is however unclear if this is simply because the larger the flash strength is the more isomerizations it causes.If this is the case, dim flashes might simply be at the resolution limit of quantification effect. In other words, does the effect scale linearly in proportion to the flash strength from dim to intermediate to strong flashes? Is so, there could be a common mechanistic origin for the phenomenon independent of flash strength. This same question is related to the discussion on pag 15.

This is a very good question and the understanding of the relation between light intensity I and OS shortening S is an important issue. Indeed, S could depend on I following a usual Michealis Menten relation, i.e.

S= (I/(I+K))

Or be closer to an all or none mechanism with a threshold, i.e. a steep logistic dependency. In order to address this issue we have looked to the shortening with a flash of light with half the intensity I used in the experiments described in Fig.5B and we have still observed a shortening (Figure 5I)

Page 17: It is hard to follow the hypothesis according to which phosphodiesterase could be responsible for the transient shortening of rods. This argument would need to be expanded and/or dropped for clarity of the presentation.

We have followed the Reviewer suggestion and we have dropped this argument at page 19.

__________________________________________________
In compliance with data protection regulations, you may request that we remove your personal registration details at any time.  (Use the following URL: <https://www.editorialmanager.com/pbiology/login.asp?a=r>). Please contact the publication office if you have any questions.
